# Metagenomic analysis of human feces reveals gut microbiome role in colorectal cancer

**DOI:** 10.3389/fcimb.2026.1828012

**Published:** 2026-06-15

**Authors:** Zehui Gu, Qi Tan, Dong Mao, Yun Zhang, Yadi Wang, Dongning He, Suxian Chen

**Affiliations:** 1 Department of Pathology, The Third Affiliated Hospital of Jinzhou Medical University, Jinzhou, Liaoning, China; 2 Department of General Surgery, The First Affiliated Hospital of Jinzhou Medical University, Jinzhou, Liaoning, China; 3 Department of Obstetrics and Gynecology, The First Affiliated Hospital of Jinzhou Medical University, Jinzhou, Liaoning, China; 4 Department of Precision Medicine, The Third Affiliated Hospital of Jinzhou Medical University, Jinzhou, Liaoning, China; 5 Department of Oncology, The Third Affiliated Hospital of Jinzhou Medical University, Jinzhou, Liaoning, China

**Keywords:** colorectal cancer, fecal biomarkers, human gut microbiome, metagenomics, early screening

## Abstract

**Background:**

This study aimed to identify the microbiota and specific genes that are closely associated with colorectal cancer (CRC) through metagenomic sequencing and integrative multi-omics analysis.

**Methods:**

Fecal samples were collected from 11 healthy volunteers and 20 patients with CRC. Genomic DNA was extracted for metagenomic analysis and high-throughput sequencing. Compositional differences and correlations of the gut microbiome were compared based on species and functional diversity.

**Results:**

The overall species composition included 1,980 species, with 1,707 species identified in the CRC group and 1,525 in the healthy control group. Alpha diversity was significantly lower in the CRC group than in the healthy control group (*p* = 0.014). Beta diversity analysis revealed significant differences between the two groups (stress = 0.1308, *p* = 0.021). Based on LEfSe analysis, *Shigella*, *Porphyromonas*, *Proteus*, *Bacteroides*, *Alistipes*, *Fusobacterium*, and *Escherichia* were more abundant in patients with CRC, whereas *Eubacterium*, *Clostridium*, *Dialister*, *Faecalibacterium*, *Blautia*, *Coprococcus*, *Dorea*, *Subdoligranulum*, *Megamonas*, *Roseburia*, *and Prevotella* were significantly more abundant in the healthy control group (*p* < 0.05).

**Conclusion:**

A multidimensional microbial diagnostic model, incorporating Shigella, Porphyromonas, Proteus, Bacteroides, Fusobacterium, Escherichia, Eubacterium, Clostridium, Dialister, Faecalibacterium, Blautia, Coprococcus, Dorea, Subdoligranulum, Megamonas, Roseburia, and Prevotella, suggests the potential to enhance early CRC screening performance. Furthermore, LptA, tnaA, envC, and argB may represent promising candidates for novel therapeutic targets, warranting further investigation.

## 1 Background

Colorectal cancer (CRC) is the third most diagnosed cancer globally and the second leading cause of cancer-related mortality, accounting for approximately 10% of all cancer cases worldwide (Capparelli et al., 2023; Sadri et al., 2024). Colonoscopy is the most effective diagnostic method; however, it requires extensive bowel preparation, involves a painful procedure, and carries risks such as iatrogenic colon perforation and mortality (Adebisi et al., 2024), leading to low acceptance among patients. Alternatively, the fecal occult blood test (FOBT; Hemoccult^®^ Test) is a widely used non-invasive CRC screening method. However, its sensitivity and specificity are limited, rendering it less effective in promptly identifying precancerous lesions. Therefore, there is an urgent need to develop more accurate and non-invasive screening methods.

The human gut is a dynamic and constantly changing ecosystem hosting trillions of microbes with essential roles in digestion, immune response, and metabolic functions, all closely linked to health and disease. Certain bacteria, such as *Bifidobacterium* and *Lactobacillus*, can be more effective in treating diseases than expensive drugs (Shah et al., 2024), which often have side effects. Disruption of the gut microbiome can contribute to cancer progression through epithelial-mesenchymal transition, angiogenesis, and metastasis to distant organs (Sevcikova et al., 2024). This highlights the intricate relationship between the gut microbiome and the occurrence and development of CRC, offering substantial potential for advancements in diagnosis and treatment.

Microbial dysbiosis is associated with various diseases, including breast, lung, liver, and pancreatic cancers (Kaźmierczak-Siedlecka et al., 2021; Esposito et al., 2022; Chen et al., 2024; Hong et al., 2024). The microbiome influences cancer development by promoting inflammation, regulating the immune system, and producing carcinogenic compounds (Saraswat and Goel, 2024). Among dysbiotic microbes, Faecalibacterium and Proteus are potentially linked to the gut microbiome. *Faecalibacterium*, one of the primary butyrate-producing bacteria in the gut, exhibits anti-inflammatory and microbiota-modulating characteristics, making it effective for treating inflammatory bowel disease, Crohn’s disease, and CRC (Song et al., 2024). As a probiotic, *Faecalibacterium* significantly reduces the frequency and formation of abnormal crypt foci in azoxymethane-induced colon cancer in rats, demonstrating its anti-tumor and anti-proliferative effects (Dikeocha et al., 2022). Furthermore, a higher relative abundance of *Faecalibacterium* is associated with a healthy gut microbiome (Antonetti et al., 2024).

*Proteus* is an opportunistic pathogen that can cause gastrointestinal, urinary tract, and diabetic foot infections (Phiri et al., 2024). *Proteus* is significantly enriched in the feces of patients with stage III–IV CRC and may contribute to CRC development (Liu et al., 2023). Genes associated with gut microbiota also play a crucial role in CRC development. The Wnt/β-catenin, MAPK/ERK, PI3K/AKT/mTOR, TGF-β, Notch, and DNA mismatch repair pathways are key in CRC pathogenesis (Fu et al., 2024; Li et al., 2024; Monge et al., 2024; Su et al., 2024; Wang et al., 2024; Nakayama et al., 2025). Traditional methods of addressing microbial dysbiosis, such as probiotics (Xu et al., 2024), aim to regulate the gut microbiome, improve gut health, and positively affect CRC. However, these approaches often fail to provide long-term benefits, raising the question of whether regulating microbiome-related genes can address the root causes of microbial dysbiosis. Therefore, it is essential to identify the genes associated with dysbiotic microbiota.

Existing literature lacks studies that systematically examine patients with CRC, differential microbiota, and differential genes as an integrated system. This study was based on CRC screening of the Chinese population and employed fecal metagenomic analysis and high-throughput sequencing to evaluate the differences and connections between beneficial and pathogenic microbes, genes, and their correlations in samples from patients with CRC and healthy controls. The aim was to identify multiple differential microbes and genes and to analyze the correlations thoroughly between microbe-microbe and microbe-gene interactions to provide new clinical biomarkers and more precise reference strategies for diagnosing and treating CRC.

## 2 Materials and methods

### 2.1 Materials

#### 2.1.1 Selection of study subjects

This study included patients who underwent endoscopic diagnosis and treatment at the Third Affiliated Hospital of Jinzhou Medical University, China. Based on pathological diagnostic results, patients with CRC were selected as the experimental group, whereas healthy volunteers were recruited as the control group. All participants met the inclusion and exclusion criteria outlined in **Table 1**, and their fecal samples were collected. Each participant signed an informed consent form, and the study was approved by the Ethics Committee of the Third Affiliated Hospital of Jinzhou Medical University. The ethics number is KX2022036.

**Table 1:** Inclusion and exclusion criteria.

	Grouping of study subjects	
	colorectal cancer patient group	healthy control group
Inclusion criteria	- Age: 40–75 years old - BMI: 18.5–30 kg/m^2^ - No history of other tumors - Histopathological diagnosis - Underwent radical resection surgery	- Age: 40–75 years old - BMI: 18.5–30 kg/m^2^
Exclusion criteria	- BMI > 30 kg/m^2^ - Non-first visit cases - Pregnancy - Lactose intolerance - Immunodeficiency - Other gastrointestinal diseases (such as inflammatory bowel disease) - Had taken antibiotics within 3 months before surgery - Infection in other parts of the body - Had taken probiotics or prebiotics within 2 weeks before surgery - Received chemotherapy or radiotherapy before surgery	BMI > 30 kg/m^2^ - Pregnancy - Lactose intolerance - Immunodeficiency - Other gastrointestinal diseases (such as inflammatory bowel disease) - Had taken antibiotics within 3 months before study - Infection in other parts of the body - Had taken probiotics or prebiotics within 2 weeks before study - Tumors in other parts of the body

*lactose intolerance introduces a confounding factor, it makes it difficult to distinguish whether the observed outcomes are caused by the experimental intervention or by the abnormal fermentation of lactose in the gut.

#### 2.1.2 Main reagents and instruments

The main instruments and reagents used were an Illumina HiSeq 2500 High-Throughput Sequencing System (Illumina, USA) and a Qiagen QIAmp DNA Stool Mini Kit (Qiagen, Germany).

### 2.2 Methods

#### 2.2.1 Collection of fecal samples and DNA quantity detection

Thirty-one participants underwent standard colonoscopy at the Third Affiliated Hospital of Jinzhou Medical University, including 20 patients with CRC and 11 healthy individuals. All participants with CRC had colonic lesions at the time of fecal collection. Fecal samples were collected and stored at −20 °C within 4 hours and at −80 °C within 24 hours for long-term preservation. DNA extraction was performed using the Qiagen QIAmp DNA Stool Mini Kit, according to the manufacturer’s instructions.

#### 2.2.2 Metagenomic analysis of gut microbiome

Raw data of 10 Gb per sample was adopted. After total DNA extraction and quality inspection of intestinal microbiota, qualified samples were sequenced on the Illumina HiSeq 2500 high-throughput sequencing platform with paired-end 150 bp sequencing mode. The obtained raw reads were subjected to quality control. FastQC was used to evaluate the quality of raw data, including the distribution of base quality (Q20/Q30), GC content and adapter contamination. Subsequently, data filtering was performed: Illumina sequencing adapter sequences were removed; bases with quality values below Q20 at both ends of reads were trimmed; sliding window scanning was applied to truncate sequences when the average quality fell below the threshold; short sequences shorter than 50 bp after trimming were discarded. High-quality clean reads were obtained for subsequent analysis. Although fecal samples are predominantly composed of bacteria, they may still contain a small amount of DNA shed from human epithelial cells. Host read removal was conducted to acquire purer microbial data. Bowtie2 was used to align qualified clean reads against the human reference genome. Reads mapped to the human genome were regarded as host contamination and eliminated, while unmapped reads were retained as non-host data for subsequent assembly and taxonomic annotation. Metagenomic assembly of clean reads was performed using MEGAHIT, and contigs shorter than 300 bp were filtered out. QUAST was used to evaluate the assembly results. MetaGeneMark (http://exon.gatech.edu/meta_gmhmmp.cgi, Version 3.26) was applied to identify coding regions in the genome. MMseqs2 (https://github.com/soedinglab/mmseqs2, Version 12-113e3) was used to remove redundant sequences. A non-redundant gene catalogue was constructed with a 95% similarity threshold and a 90% coverage threshold. Functional annotation and taxonomic analysis of the non-redundant gene catalogue were performed across multiple databases, and the composition and abundance of species in samples were statistically analyzed. All analyzes were completed based on BMKCloud (http://www.biocloud.net/). Multiple hypothesis testing correction was conducted to control the false positive rate in inter-group differential analyzes (differential species, differential genes and differential pathways). The Benjamini-Hochberg method was used for P-value correction in multiple comparisons, and differences with FDR<0.05 were considered statistically significant. During this phase, samples were excluded due to insufficient DNA purity, inadequate sequencing depth (insufficient valid reads), or poor reproducibility. Ultimately, 22 samples were retained for subsequent metagenomic analysis.

Species composition analysis was performed to determine the relative abundance of dominant species between groups. Alpha diversity analysis was performed to assess the species or functional richness and diversity between the two groups. Beta diversity analysis, including principal component analysis (PCA), principal coordinate analysis (PCoA), and non-metric multidimensional scaling (NMDS), was used to examine differences in species or functional dimensions between the two groups. LEfSe analysis was performed to identify biomarkers in species between the two groups, with a linear discriminant analysis score threshold of 3.0. Random forest analysis was used to rank important species features between the two groups. Additionally, the non-redundant gene set sequences were aligned with the Kyoto Encyclopedia of Genes and Genomes (KEGG) and the Evolutionary genealogy of genes: Non-supervised Orthologous Groups (eggNOG) databases for functional annotation and beta diversity analysis to identify differential functional annotations between the two groups. The corresponding tools from the Carbohydrate-active enzymes (CAZy) and the comprehensive Antibiotic Research Database (CARD) were used to compare and analyze differences in carbohydrate-active enzymes and antibiotic resistance, respectively.

### 2.3 Statistical analysis

Statistical analysis was performed using SPSS version 26.0 software. Categorical data were presented as frequencies (percentages). Group comparisons were performed using the T-test or Fisher’s exact probability test, and independent sample comparisons were performed using the two-tailed Wilcoxon rank-sum test (Mann–Whitney U-test). The alpha diversity of the bacterial communities was calculated using the R vegan package and represented using Simpson and Shannon indices. Similarity analysis between intestinal tissues and fecal samples was performed using the binary Jaccard and Bray–Curtis dissimilarity models. Species analysis was performed using analysis of similarities(ANOSIM) and permutational multivariate analysis of variance (PERMANOVA) functions with 1,000 permutations. Statistical significance was set at p < 0.05.

## 3 Results

### 3.1 Overall comparison of fecal microbiota between healthy individuals and patients with CRC

**Figure 1:**
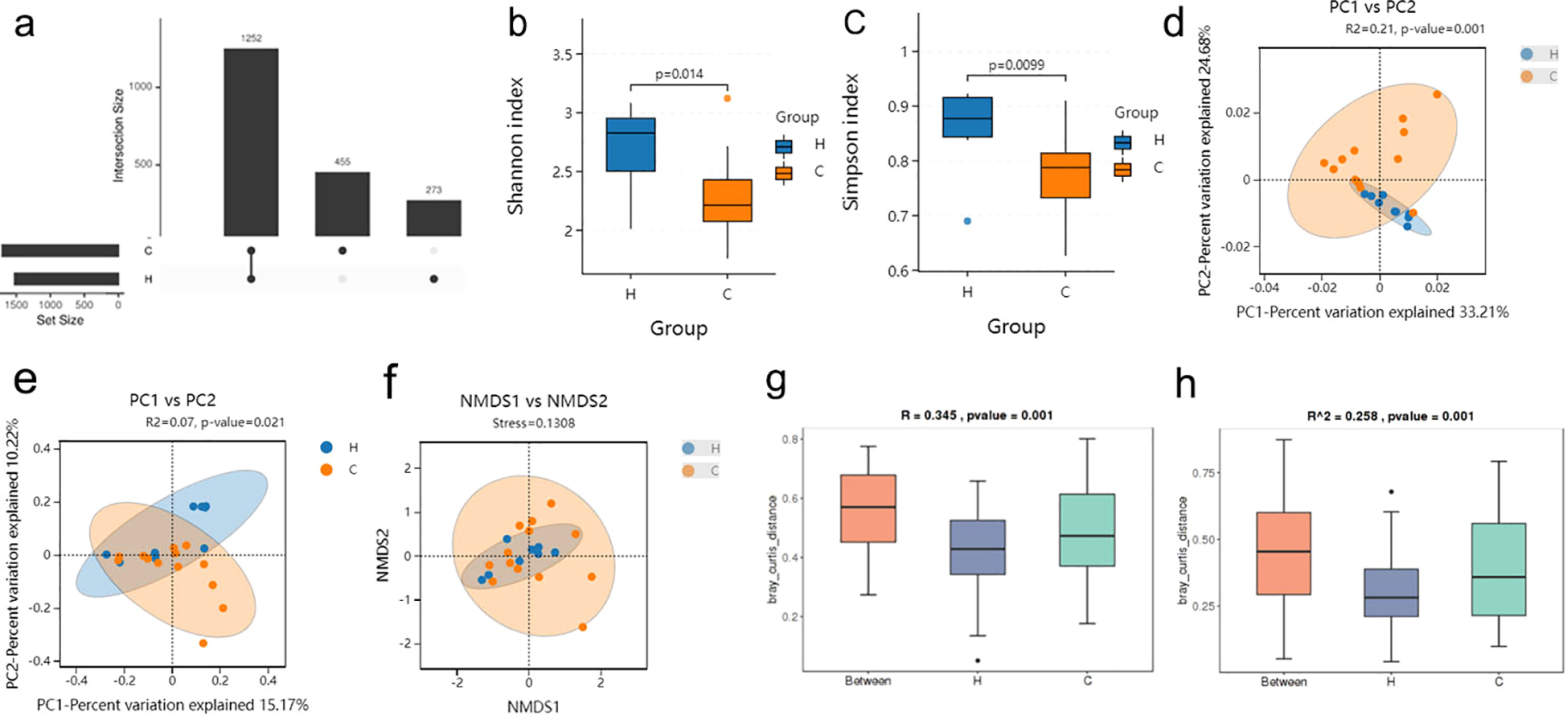
Overview of gut microbiome profiles in healthy control (H) and CRC (C) groups. **(a)** UpSet plot of species composition, with a side bar graph showing the number of species included in each group. The connections between dots represent overlaps, similar to a traditional Venn diagram. **(b)** Shannon diversity index difference analysis plots showed that the healthy control group (H) index was significantly higher than that of the CRC group (*p* = 0.014). **(c)** Simpson diversity index difference analysis plots showed that the healthy control group (H) index was significantly higher than that of the CRC group (*p* = 0.0099). **(d)** The species abundance PCA plot shows points representing individual samples, with different colors indicating different groups. The x-axis represents the first principal component and its contribution to the sample differences, whereas the y-axis represents the second principal component and its contribution to the sample differences (R^2^ = 0.21, *p* = 0.001). **(e)** The PCoA plot of species abundance shows the x- and y-axes as the two eigenvalues that contribute the most to the sample differences, which are expressed as percentages to indicate the degree of their influence (R^2^ = 0.07, *p* = 0.021). **(f)** The NMDS analysis plot of species shows differences in the overall fecal microbiota composition between the healthy control (H) and CRC groups (stress = 0.1308, *p* = 0.021). **(g)** Boxplot of inter-sample distances for species (ANOSIM analysis) indicates greater differences between groups than within groups (R = 0.345, *p* = 0.001). **(h)** The boxplot of inter-sample distances for species (PERMANOVA) provides an R^2^ value, which indicates the extent to which the different groupings explain the sample differences (R^2^ = 0.258, *p* = 0.001).

We assessed the gut microbiome in fecal samples from 22 subjects, consisting of 1,980 operational taxonomic units (OTUs), with 1,707 OTUs in the CRC group and 1,525 OTUs in the healthy control group, most of which were shared between the groups (**Figure 1a**). Using Shannon and Simpson indices to measure microbial richness and diversity levels, Alpha diversity analysis revealed that the CRC group had significantly lower Shannon and Simpson diversity indices than the control group (*p* = 0.014 and *p* = 0.0099, respectively; **Figures 1b, c**). This primarily highlights the core characteristics of microbial structural disorder and ecological imbalance. Beta diversity between the two groups was examined by mapping the distance differences between the two groups using PCA and PCoA, which showed significant clustering between the groups (R^2^ = 0.21, *p* = 0.001; R^2^ = 0.07, *p* = 0.021, **Figures 1d, e**). NMDS was used to compare beta diversity between the CRC and control groups, which revealed significant differences between the two groups in the NMDS1 and NMDS2 dimensions (stress = 0.1308, *p* = 0.021, **Figure 1f**). Furthermore, ANOSIM and PERMANOVA revealed significant differences between the fecal samples of the two groups (R = 0.345, *p* = 0.001; R^2^ = 0.258, *p* = 0.001, **Figures 1g, h**). These findings suggest that during CRC progression, microbial alterations are not merely local or subtle fluctuations. Instead, the microbiota undergoes a multidimensional transformation—a systemic ‘collapse’ followed by ‘remodeling’—This process presents a possibility for clinical diagnosis that warrants further exploration.

### 3.2 Fecal composition analysis in healthy individuals and patients with CRC

**Figure 2:**
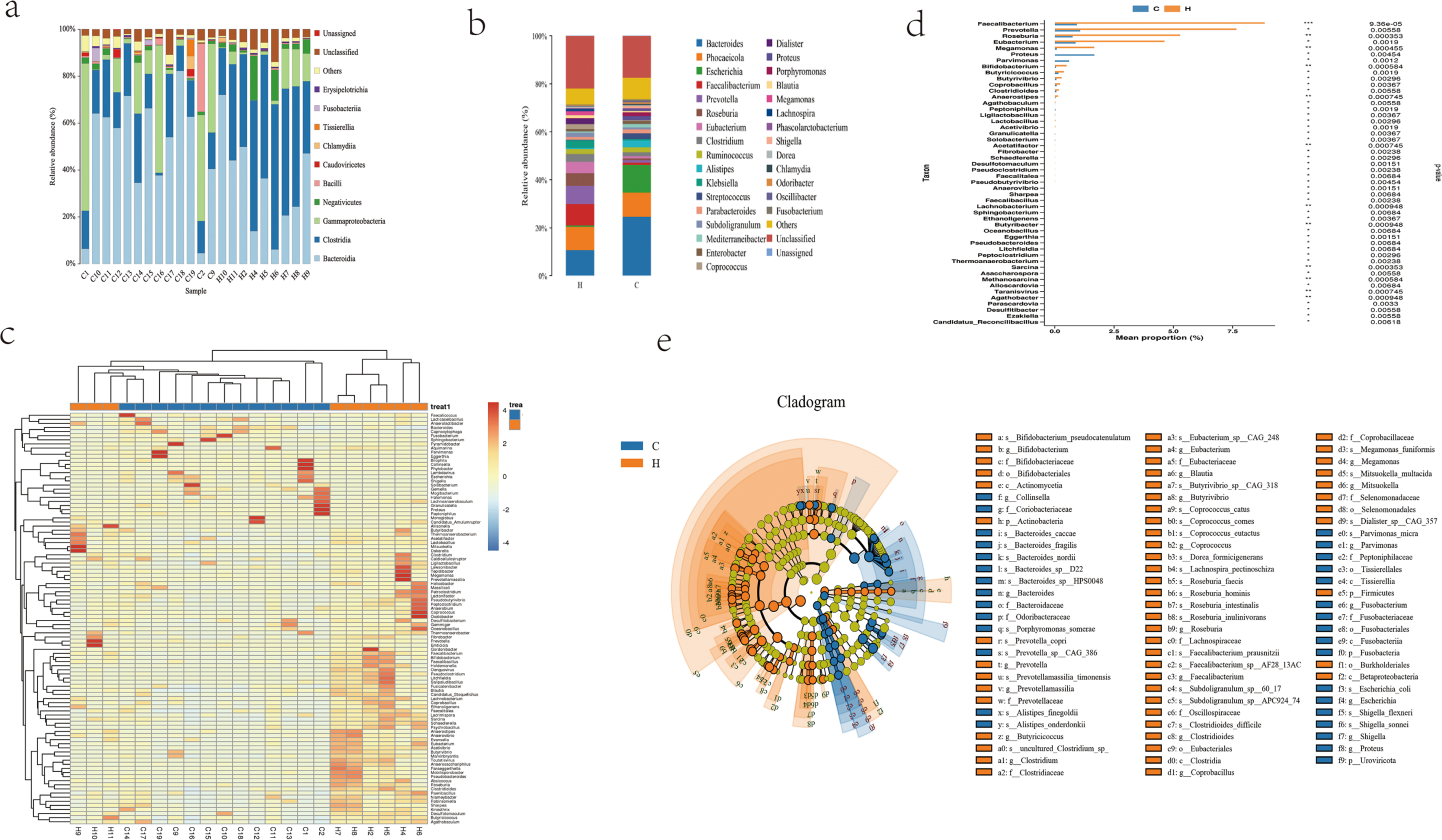
Fecal composition analysis of healthy control (H) and CRC (C) groups. **(a)** Relative abundance plot of bacterial phyla in fecal samples from groups H and C. **(b)** Abundance plot of the top 30 bacterial genera with the highest relative expression levels in each group of samples. **(c)** A heatmap of differential species abundance; the left dendrogram shows the hierarchical clustering of species, the upper dendrogram displays the hierarchical clustering of samples, and the central heatmap illustrates the abundance of species. **(d)** The histogram of differential species abundance shows the mean abundance of species on the vertical axis. The top 50 species were ranked by p-values in descending order and sorted by abundance from high to low. Significant differences were observed between the two groups for the bacterial genera, including *Faecalibacterium*, *Prevotella*, *Roseburia*, *Eubacterium*, *Megamonas*, and *Proteus*. **(e)** LEfSe evolutionary branching diagram with the circles radiating outward, representing the taxonomic levels from kingdom to species. The diameter of the small circle is proportional to the relative abundance; different colors represent different groups. Nodes of different colors indicate microbes that play an important role within the respective groups.

Taxonomic composition analysis of the fecal samples from the two groups revealed that the collected sequences were primarily composed of six phyla: Bacteroidetes, Firmicutes, Proteobacteria, Actinobacteria, Uroviricota, and Chlamydiae (**Figure 2a**). At the genus level, based on the compositional makeup of the samples in the two groups, the top 30 bacterial genera were more abundant (**Figure 2b**). A significance test for the top-ranked bacterial genera revealed significant differences between the groups, with *Faecalibacterium*, *Prevotella*, *Roseburia*, *Eubacterium*, *Megamonas*, and *Proteus* showing differences in patients with CRC compared to those in the healthy control group (*p* < 0.05, **Figures 2c, d**). High-dimensional comparisons using LEfSe indicated that patients with CRC had higher expression levels of *Shigella* (*p* = 0.025), *Porphyromonas* (*p* = 0.041), *Proteus* (*p* = 0.004), *Bacteroide*s (*p* = 0.016), *Alistipes* (*p* = 0.012), *Fusobacterium* (*p* = 0.030), and *Escherichia* (*p* = 0.042), whereas the healthy control group had higher expression levels of *Eubacterium* (*p* = 0.002), *Clostridium* (*p* < 0.005), *Dialister* (*p* = 0.033), *Faecalibacterium* (*p* = 9.36E−05), *Blautia* (*p* = 0.010), *Coprococcus* (*p* = 0.012), *Dorea* (*p* = 0.008), *Subdoligranulum* (*p* = 0.035), Megamonas (*p* < 0.005), Roseburia (*p* = 0.002), and *Prevotella* (*p* = 0.018) (**Figure 2e**). The differentiation of dominant genera may suggest not only quantitative changes in bacterial abundance, but also potentially indicate a systemic shift within the gut microbiome.

### 3.3 Species differences and correlation between healthy individuals and patients with CRC

**Figure 3:**
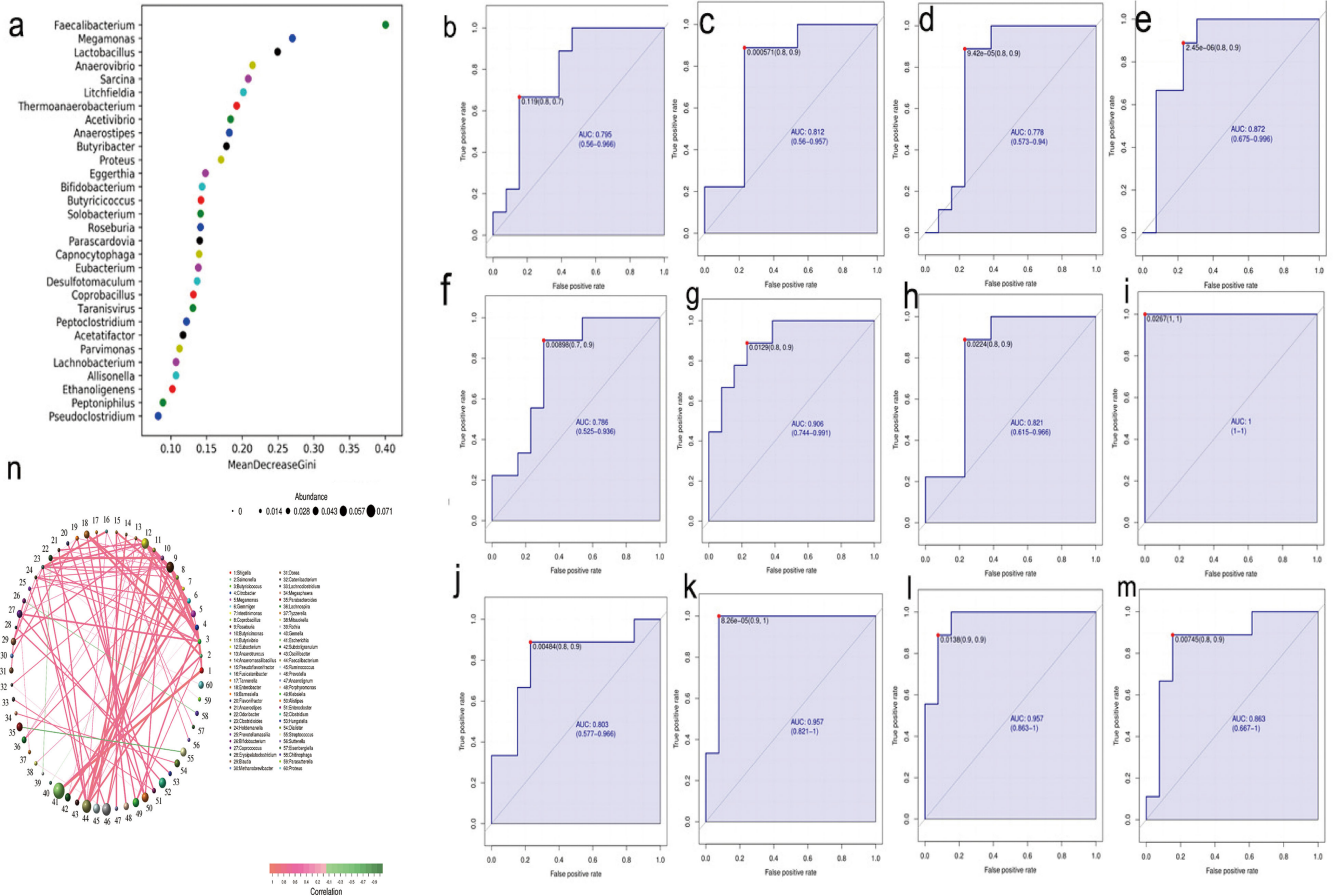
Species differences and correlations between the groups. **(a)** Random forest analysis of species importance ranking plot; the x-axis represents the species importance measure. Higher values indicate a greater decrease in classification accuracy when the species is removed, highlighting its importance in sample classification. The y-axis represents the species names ranked by importance. **(b–m)** Receiver operating characteristic curve, with the performance evaluated based on the area under the curve. **(n)** Species correlation network diagram, where circles represent species, and their size is proportional to species abundance. Lines connecting the circles represent correlations between species, and the thickness of the lines indicates the strength of the correlation. Line colors denote the type of correlation: red represents a positive correlation; green represents a negative correlation.

The random forest algorithm was employed to construct multiple decision trees to classify samples and identify microbial species that significantly contributed to the differences between samples. The resulting species importance ranking plot showed that *Faecalibacterium emerged as a promising candidate feature species in this preliminary analysis.* (**Figure 3a**). Given the small sample size, the model parameters were directly optimized using 10-fold cross-validation to enhance the accuracy of the model. Receiver operating characteristic curve analysis showed that *Bacteroides* (area under the curve [AUC] = 0.795), *Shigella* (AUC = 0.812), *Fusobacterium* (AUC = 0.778), *Proteus* (AUC = 0.872), *Escherichia* (AUC = 0.786), *Eubacterium* (AUC = 0.906), *Clostridium* (AUC = 0.821), *Faecalibacterium* (AUC = 1.000), *Blautia* (AUC = 0.829), *Coprococcus* (AUC = 0.803), *Megamonas* (AUC = 0.957), *Roseburia* (AUC = 0.957), and *Prevotella* (AUC = 0.863). These microorganisms demonstrate the potential to predict CRC diagnosis. (**Figures 3b–m**).

To explore microbial associations, we selected 80 species with the highest abundance and performed correlation analysis based on their abundance and variation in the samples. Using the Spearman algorithm (including positive and negative correlations) and statistical tests, data sets with correlations greater than 0.5 and p < 0.05 were selected. A correlation network diagram was generated using R. Significant positive correlations were observed between the following species: *Alistipes*, *Bucyricimonas*, and *Odoribacter*; *Shigella* and *Escherichia*; *Eubacterium*, *Faecalibacterium*, *Blautia*, *Roseburia*, and *Lachnospira*; *Clostridium* and *Coprococcus*; *Blautia* and *Dorea*; *Faecalibacterium*, *Roseburia*, and *Megamonas*; *Megamonas* and *Roseburia*; and *Roseburia* and *Prevotella* (*p* < 0.005, **Figure 3n**). These findings unveil the intricate ‘social network’ and ‘functional alliances’ within the gut microecosystem. They suggest that therapeutic interventions should extend beyond targeting single species to focusing on the holistic status of these ‘microbial modules.’ Restoring symbiotic relationships may hold greater promise than single-bacteria supplementation, which could offer valuable insights for clinical therapeutic strategies.

### 3.4 Overall comparison of functional changes in fecal microbiota associated with CRC

**Figure 4:**
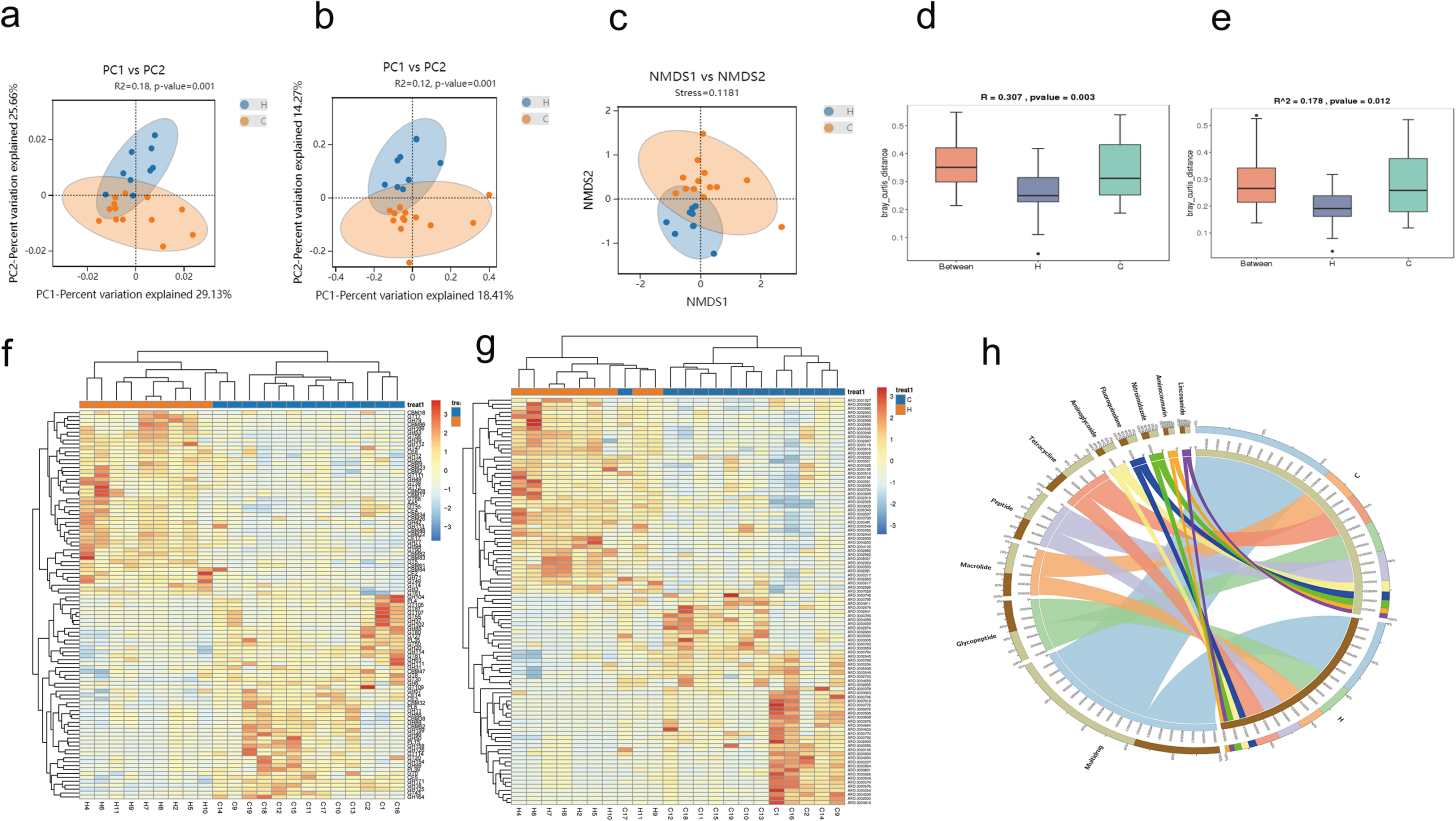
Functional gut microbiome changes in healthy control (H) and CRC (C) groups. **(a)** PCA analysis of functional genes (R^2^ = 0.18, *p* = 0.001). **(b)** PCoA analysis of functional gene abundance (R^2^ = 0.12, *p* = 0.001). **(c)** NMDS analysis of functional genes (stress = 0.1181, *p* = 0.001). **(d)** ANOSIM plot of inter-sample distances for functional genes shows that the analysis indicates greater differences between groups than within groups (R = 0.307, *p* = 0.003). **(e)** PERMANOVA analysis of inter-sample distances for functional genes (R^2^ = 0.178, *p* = 0.012). **(f)** Heatmap of differential carbohydrate-active enzyme abundance showing the clustering tree of differential enzymes on the left, the sample clustering tree at the top, and the heatmap in the center. **(g)** Heatmap of the differential abundance of antibiotic resistance ontology. **(h)** Circle plot of CARD for detecting the antibiotic resistance gene composition, with the outer right half-circle representing the samples and the left half-circle representing the annotated resistance gene types. Scale represents the proportion of abundance. The inner ribbons connect the resistance genes and samples, revealing the functional composition and distribution of resistance genes in the samples. The width of the ribbons represents the proportional distribution.

To characterize microbial gene functions and the differences between patients with CRC and non-tumor participants, we quantified the relative abundances of prokaryotic genes using KEGG, eggNOG, the pathogen-host interaction database (PHI-base), and metagenomic gene modules in the study samples. For beta diversity analysis, differences between the two groups were mapped using PCA and PCoA analyzes. Significant clustering was observed between groups (R^2^ = 0.18, *p* = 0.001; R^2^ = 0.12, *p* = 0.001; **Figures 4a, b**). NMDS analysis of beta distances between the two groups revealed significant differences in NMDS1 and NMDS2 dimensions (stress = 0.1181, *p* = 0.001, **Figure 4c**). Moreover, ANOSIM and PERMANOVA analyzes indicated significant differences between the two groups in fecal samples (R = 0.307, *p* = 0.003; R^2^ = 0.178, *p* = 0.012, **Figures 4d, e**). To investigate the carbohydrate utilization preferences of the microbiota and functional genes associated with antibiotic resistance, the metagenome was annotated using prokaryotic carbohydrate-active enzyme families from the CAZy database and antibiotic resistance families from the CARD database. The results showed significant differences between the two groups, with 42 carbohydrate-active enzymes, 38 antibiotic resistance ontologies, and nine antibiotic resistance genes that were significantly abundant in patients with CRC (*p* < 0.005, **Figures 4f–h**). In summary, these preliminary findings suggest that the intestinal environment of patients with colorectal cancer (CRC) may undergo systemic functional alterations. These observations could provide preliminary clues for subsequent exploration of its pathogenic mechanisms and the development of precision therapies, highlighting the potential exploratory value of this research in translational medicine.

### 3.5 Differences in the microbial-related metabolic composition between fecal samples of patients with CRC and healthy individuals

**Figure 5:**
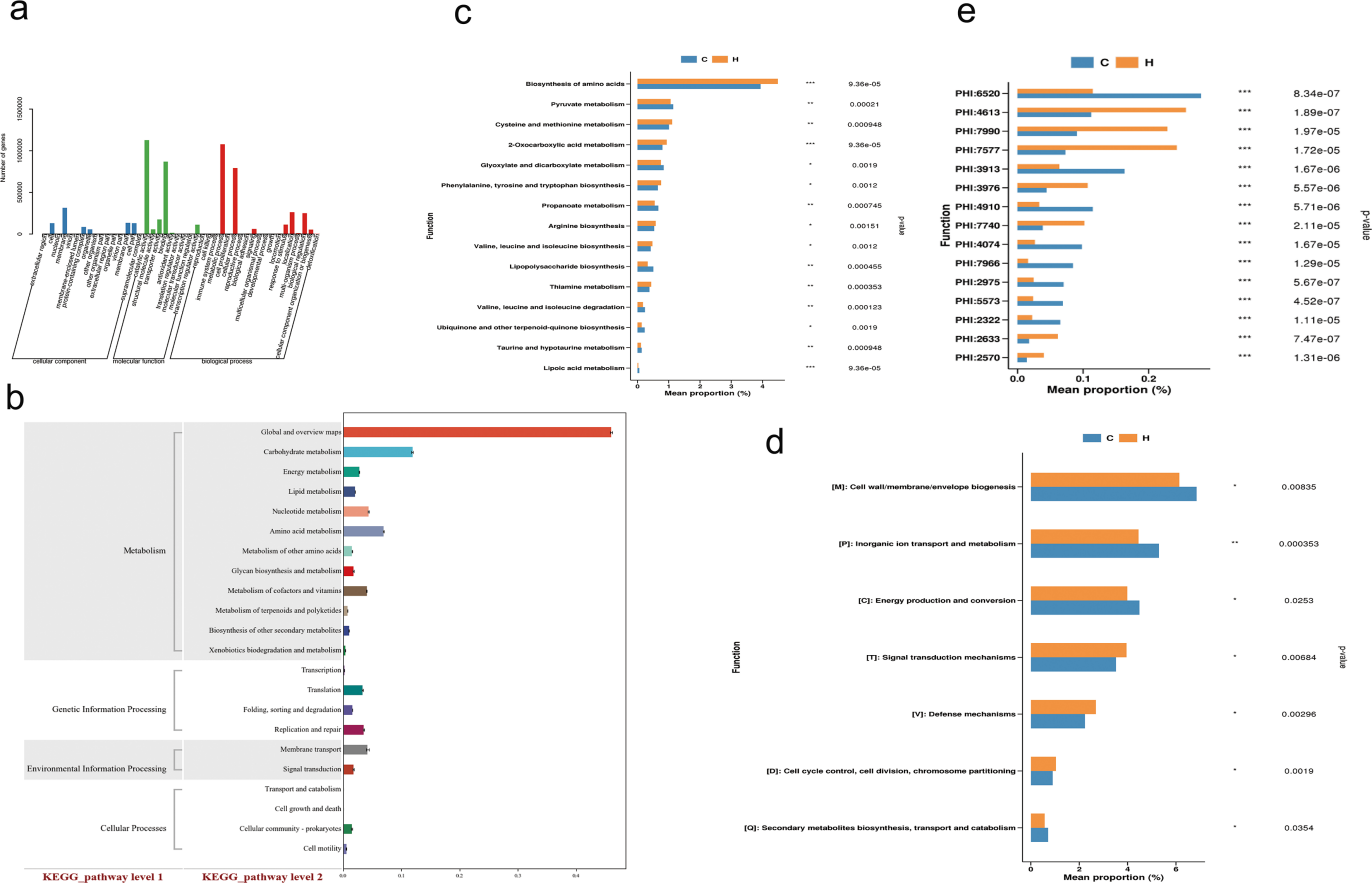
Differential metabolic composition between groups. **(a)** Statistical plot of GO secondary node annotation classification, with the horizontal axis representing the GO category content and the vertical axis (on the left) showing the number of genes. **(b)** Statistical plot of KEGG metabolic pathway-related functional genes at level 2, with the horizontal axis showing the relative abundance of the corresponding functional genes and the vertical axis representing the classification content of KEGG levels 1 and 2. **(c)** Histogram of the KEGG pathway (level 3) showing the differential functional genes, with the leftmost axis indicating the mean functional abundance, the horizontal axis representing the mean proportion, and the vertical axis representing the functional names. **(d)** eggNOG (class-level) histogram shows the differential functional genes between the groups. **(e)** Histogram of PHI-base demonstrating the differential functional genes between the groups, where * indicates *p* < 0.05, ** indicates *p* ≤ 0.001, and *** indicates *p* ≤ 0.0001. The p-values are displayed on the rightmost side, with the data ordered by descending p-value (top 15 selected) and then by abundance, from high to low.

Gene ontology (GO) is a database that defines and describes gene and protein functions. Based on the GO functional annotations, the top enriched GO terms were primarily related to biological processes (e.g., metabolic process, cellular process, localization, and biological regulation), cellular components (e.g., membrane, cell, and membrane part), and molecular functions (e.g., catalytic activity, binding, and transporter activity) (**Figure 5a**). KEGG is a comprehensive database that collects genomic, pathway, and compound data from various organisms. Orthologous protein groups were formed by clustering sequences using KEGG. The most abundant enriched pathways in the KEGG orthologous protein groups were associated with global and overview maps, carbohydrate metabolism, nucleotide metabolism, amino acid metabolism, replication and repair, membrane transport, and translation (**Figure 5b**).

Intergroup differences between the control and CRC groups were analyzed based on the KEGG pathway (Level 3). Significant differences were observed in pathways such as amino acid biosynthesis, pyruvate metabolism, cysteine and methionine metabolism, 2-oxoacid metabolism, hydroxymethylglutaryl-CoA metabolism, and diphenylmethane ester metabolism (*p* < 0.05, **Figure 5c**). Additionally, the eggNOG database contains orthologous gene clusters from organisms, with proteins in each cluster assumed to originate from an ancestral protein with the same function. According to eggNOG annotations (class level), significant differences were found between the healthy control and CRC groups in processes such as cell wall/membrane/envelope biogenesis, inorganic ion transport and metabolism, energy production and conversion, signal transduction mechanisms, defense mechanisms, cell cycle control, cell division, chromosome partitioning, and the biosynthesis, transport, and degradation of secondary metabolites (*p* < 0.05, **Figure 5d**). The PHI-base includes experimentally validated gene data, primarily from fungi, oomycetes, and bacterial pathogens. The infected hosts include animals, plants, fungi, and insects. Based on PHI-base annotations, significant differences were observed between the two groups in genes such as PHI:6520 (*LpdA*), PHI:4613 (*esaN*), PHI:7990 (*LysX*), PHI:7577 (*cstA*), PHI:3913 (*SpMsrAB2*), PHI:3976 (*MoIlv6*), PHI:4910 (*LptA*), PHI:7740 (*graS*), PHI:4074 (*wecA*), PHI:7966 (*tnaA*), PHI:2975 (*ipx10*), PHI:5573 (*envC*), PHI:2322 (*SidH*), PHI:2633 (*argB*), and PHI:2570 (*CYB2*) (*p* < 0.05, **Figure 5e**), with *LptA*, *tnaA*, *envC*, and *argB* being closely related to *Escherichia coli*. In summary, this approach demonstrates potential for clinical diagnosis. It offers new insights into exploring the pathogenic mechanisms of colorectal cancer (CRC) and developing precision therapies, highlighting its exploratory significance in translational medicine research.

## 4 Discussion

CRC is one of the malignancies with the highest incidence and mortality rate worldwide. Commonly used early screening methods for CRC include FOBT, colonoscopy, and colon computed tomography imaging. However, each screening method has limitations. With advances in microbial community research, human fecal metagenomic sequencing has emerged as a sophisticated, non-invasive method for early CRC screening. This approach directly reflects the ecological status of the gut and aids in exploring the pathogenesis of various chronic diseases, including cancer. Research on CRC has yet to identify definitive carcinogenic pathogens similar to those found in gastric, cervical, and liver cancers. Abnormal indicators of CRC from species and functional perspectives require elucidation to identify precise diagnostic and therapeutic biomarkers. Fecal biomarker detection is expected to enhance the convenience, sensitivity, and clinical practicality of screening and improve patient compliance.

Increasing evidence (Wang et al., 2023) suggests that the gut microbiome is closely related to the onset and progression of CRC. Our metagenomic sequencing data suggest potential differences in the dominant and subordinate genera between the two groups. These findings are consistent with those of previous studies by Cao et al. and Kushkevych et al (Cao et al., 2021; Kushkevych et al., 2024), indicating that the gut microbiome associated with CRC has a higher species richness and a greater abundance of pathogenic microbes. However, these studies primarily focused on ulcerative colitis or rat models, whereas our study involved individuals with colonic lesions, providing a more representative sample. In addition, correlation analysis was performed on highly abundant species to identify significant associations within the abnormal gut microbiome. We observed that, from a biological perspective, these bacteria likely share similar ecological niches, exhibit metabolic synergism, or are subject to common regulation by the host’s diet and immune status. A high-fat diet can influence *Alistipes* and *Odoribacter*, leading to the production of novel metabolic products, including sulfonate compounds, which are closely linked to inflammatory bowel disease through the Toll-like receptor 4 (TLR4) signaling pathway (Walker et al., 2017; Older et al., 2023). *Shigella* and *Escherichia* are key microbes for food safety and clinical diagnostics. Genomic deletions can enhance the virulence of Shigella spp. and enteroinvasive Escherichia coli, increasing their pathogenicity (Maurelli et al., 1998; Liu et al., 2024). *Eubacterium*, *Faecalibacterium*, *Blautia*, *Roseburia*, and others can produce short-chain fatty acids (such as butyrate), which play important roles in immune regulation and preventing early CRC (Vacca et al., 2020; Gao et al., 2022). Changes in the gut microbiome of *Clostridium* sp. *BR31* and *Coprococcus 1* strengthen the relationship between dietary imbalance and gastrointestinal symptoms in children with autism spectrum disorder (Li et al., 2023). The correlation between *Faecalibacterium* and *Megamonas* in the gut microbiome is complex. A study in Henan showed a positive correlation between *Faecalibacterium* and *Megamonas* species. Other studies (Lai et al., 2024) have also suggested that various factors, including dietary habits, living environment, and genetic factors, influence the relative abundance of *Faecalibacterium* and *Megamonas* in the gut microbiome. These studies are consistent with our findings, although correlations between some microbiota have not been documented in the literature. It is undeniable that Faecalibacterium plays a significant role within the gut microbiota. However, while we employed rigorous cross-validation strategies to assess model performance, the AUC of 1.000 obtained in the context of a limited sample size suggests a potential risk of overfitting. The absence of an independent test set implies that the model’s generalizability has not yet been substantiated. Therefore, caution should be exercised when interpreting the potential of Faecalibacterium as a biomarker, and future studies should focus on validating this finding in independent cohorts.

Changes at the genetic level may influence microbial alterations and immune status in CRC. We identified genes associated with the gut microbiome, these genes exhibit varying degrees of association with the gut microbiome. For example, LptA, tnaA, envC, and argB are closely linked in E. coli. LptA is a periplasmic lipid A-binding protein involved in the lipopolysaccharide export pathway (Tran et al., 2008). Disruption of the LptA/LptC interaction inhibits the growth of Gram-negative bacteria. Moreover, *tnaA* encodes tryptophanase, an enzyme widely found in gram-negative bacteria but is present in only a few gram-positive bacteria (Boya et al., 2021). Transcription and translation occur simultaneously in E. coli, a prokaryote without a membrane-bound nucleus, resulting in a high demand for tryptophan. Enhancing key genes, such as *trpR*, *tnaA*, *aroG*, and *trpED*, increases tryptophan production. In addition, *envC* encodes a cell wall hydrolase activator in *E. coli*.

In Gram-negative bacteria, EnvC promotes cell separation and division by activating cell wall hydrolases via interactions with the FtsEX complex (Xu et al., 2023). *argB* encodes acetylglutamate kinase in *E. coli*, which catalyzes the second step in the biosynthesis of ornithine and arginine. Ornithine and arginine may help maintain the gut microbial balance and reduce the growth and infection of harmful bacteria such as *E. coli*. Recent studies have revealed that colorectal cancer (CRC) cells universally exhibit a phenotype of “arginine auxotrophy.” This metabolic vulnerability stems from the downregulation of argininosuccinate synthase (the mammalian homolog of the bacterial argB gene), which renders the cells incapable of *de novo* arginine biosynthesis (Xu et al., 2025). Consequently, CRC cells appear to become dependent on exogenous arginine uptake, an observation that further supports the potential biological characteristics associated with argB deficiency. However, no study has directly discussed the relationship between argB and *E. coli*. Our findings suggest that LptA, tnaA, and envC levels are elevated in CRC, whereas argB levels are reduced. This observation has not been previously reported. The current treatment options for CRC include endoscopic resection, surgery, chemotherapy, radiotherapy, targeted therapy, and immunotherapy. Given the close relationship between microbes and genes, genetic intervention may emerge as a promising new therapeutic approach.

In summary, human fecal metagenomic analysis and high-throughput sequencing through an integrative multi-omic analysis were employed to identify the microbiota and specific genes closely associated with CRC, enabling a comprehensive study. Significant differences were observed between the healthy control and CRC groups, and microbe-to-microbe and microbe-to-gene correlations were explored. We have initial constructed a multidimensional microbial diagnostic model incorporating Shigella, Porphyromonas, Proteus, Bacteroides, Alistipes, Fusobacterium, Escherichia, Eubacterium, Clostridium, Dialister, Faecalibacterium, Blautia, Coprococcus, Dorea, Subdoligranulum, Megamonas, Roseburia, and Prevotella. This model significantly enhances the sensitivity and specificity of early CRC screening. Furthermore, our preliminary exploration identifies LptA, tnaA, envC, and argB as promising novel therapeutic targets, which also hold potential as predictive biomarkers for CRC. This study highlights the advantages of combining microbiome research with CRC screening and treatment, offering new perspectives for identifying key carcinogenic pathogens in CRC. From the perspective of tumor staging, all enrolled CRC patients were classified as Stage I or II. This characteristic suggests that the constructed composite diagnostic model, along with the preliminary gene targets identified, holds potential clinical value for the early screening of colorectal cancer. However, this study also has limitations that limit the depth of the research. A small number of cases may not allow for more detailed studies such as stratified analysis. For example, it is difficult to stratify patients according to the severity of the disease and study treatment effects, making it difficult to comprehensively understand the relationship between the disease and treatment methods. Future studies will be dedicated to expanding the sample cohort and refining subject stratification. We aim to extend our scope beyond CRC patients to include individuals with enteritis, intestinal polyps, and tubular adenomas, thereby systematically characterizing the gut microbiota landscape across different stages of disease progression. Concurrently, we will investigate the expression profiles of Escherichia coli-associated genes to gain deeper insights into the mechanisms underlying disease pathogenesis. With advances in healthcare, new screening technologies and treatment methods will provide patients with more options and better treatment outcomes. However, challenges remain regarding the prevention and treatment of CRC. Therefore, there is a need to improve early diagnosis rates and strengthen public health education to increase awareness and prevent CRC.

## Data Availability

The datasets presented in this study can be found in online repositories. The names of the repository/repositories and accession number(s) can be found in the article/supplementary material.

## References

[B3] Adebisi A. A. Onobun D. E. Adediran A. Ononye R. N. Ojo E. O. Oluyi A. . (2024). Evaluation of morbidity and mortality in iatrogenic colonic perforation during colonoscopy: a comprehensive systematic review and meta-analysis. Cureus 16, e73302. doi: 10.7759/cureus.73302 39655125 PMC11625968

[B13] Antonetti L. Berrilli F. Di Cristanziano V. Farowski F. Daeumer M. Eberhardt K. A. . (2024). Investigation of gut microbiota composition in humans carrying blastocystis subtypes 1 and 2 and Entamoeba hartmanni. Gut Pathog. 16, 72. doi: 10.1186/s13099-024-00661-5 39614306 PMC11607961

[B35] Boya B. R. Kumar P. Lee J. H. Lee J. (2021). Diversity of the tryptophanase gene and its evolutionary implications in living organisms. Microorganisms 9, 2156. doi: 10.3390/microorganisms9102156 34683477 PMC8537960

[B24] Cao H. Zong C. Dai W. Gao Q. Li D. Wu X. . (2021). The effects of Chinese medicine QRD, antibiotics, and probiotics on therapy and gut microbiota in septic rats. Front. Cell. Infect. Microbiol. 11, 712028. doi: 10.3389/fcimb.2021.712028 34722329 PMC8552555

[B2] Capparelli R. Cuomo P. Gentile A. Iannelli D. (2023). Microbiota-liver diseases interactions. Int. J. Mol. Sci. 24, 3883. doi: 10.3390/ijms24043883 36835291 PMC9959879

[B8] Chen P. Yang C. Ren K. Xu M. Pan C. Ye X. . (2024). Modulation of gut microbiota by probiotics to improve the efficacy of immunotherapy in hepatocellular carcinoma. Front. Immunol. 15, 1504948. doi: 10.3389/fimmu.2024.1504948 39650662 PMC11621041

[B12] Dikeocha I. J. Al-Kabsi A. M. Chiu H. T. Alshawsh M. A. (2022). Faecalibacterium prausnitzii ameliorates colorectal tumorigenesis and suppresses proliferation of HCT116 colorectal cancer cells. Biomedicines 10, 1128. doi: 10.3390/biomedicines10051128 35625865 PMC9138996

[B6] Esposito M. V. Fosso B. Nunziato M. Casaburi G. D'Argenio V. Calabrese A. . (2022). Microbiome composition indicate dysbiosis and lower richness in tumor breast tissues compared to healthy adjacent paired tissue, within the same women. BMC Cancer 22, 30. doi: 10.1186/s12885-021-09074-y 34980006 PMC8722097

[B17] Fu J. Zhou L. Li S. Zheng J. Hou Z. He P. (2024). Let-7c-5p down regulates the proliferation of colorectal cancer through the MAPK-ERK-signaling pathway. Biochem. Genet. 62, 3231–3243. doi: 10.1007/s10528-023-10581-9 38095736

[B30] Gao R. Wu C. Zhu Y. Kong C. Zhu Y. Gao Y. . (2022). Integrated analysis of colorectal cancer reveals cross-cohort gut microbial signatures and associated serum metabolites. Gastroenterology 163, 1024–1037.e9. doi: 10.1053/j.gastro.2022.06.069 35788345

[B7] Hong R. Lin S. Zhang S. Yi Y. Li L. Yang H. . (2024). Pathogen spectrum and microbiome in lower respiratory tract of patients with different pulmonary diseases based on metagenomic next-generation sequencing. Front. Cell. Infect. Microbiol. 14, 1320831. doi: 10.3389/fcimb.2024.1320831 39544279 PMC11560916

[B9] Kaźmierczak-Siedlecka K. Stachowska E. Folwarski M. Przewłócka K. Makarewicz W. Bryl E. (2021). The potential of gut microbiome as a non-invasive predictive biomarker for early detection of pancreatic cancer and hepatocellular carcinoma. Eur. Rev. Med. Pharmacol. Sci. 25, 7275–7284. doi: 10.26355/eurrev_202112_27421 34919227

[B25] Kushkevych I. Martínková K. Mráková L. Giudici F. Baldi S. Novak D. . (2024). Comparison of microbial communities and the profile of sulfate-reducing bacteria in patients with ulcerative colitis and their association with bowel diseases: a pilot study. Microb. Cell. 11, 79–89. doi: 10.15698/mic2024.03.817 38486888 PMC10939707

[B33] Lai J. Gong L. Liu Y. Zhang X. Liu W. Han M. . (2024). Associations between gut microbiota and osteoporosis or osteopenia in a cohort of Chinese Han youth. Sci. Rep. 14, 20948. doi: 10.1038/s41598-024-71731-6 39251661 PMC11385745

[B32] Li H. Liu C. Huang S. Wang X. Cao M. Gu T. . (2023). Multi-omics analyses demonstrate the modulating role of gut microbiota on the associations of unbalanced dietary intake with gastrointestinal symptoms in children with autism spectrum disorder. Gut Microbes 15, 2281350. doi: 10.1080/19490976.2023.2281350 38010793 PMC10730204

[B18] Li Z. Ke H. Cai J. Ye S. Huang J. Zhang C. . (2024). MTHFD1 regulates autophagy to promote growth and metastasis in colorectal cancer via the PI3K-AKT-mTOR signaling pathway. Cancer Med. 13, e70267. doi: 10.1002/cam4.70267 39571599 PMC11581708

[B15] Liu J. Huang X. Chen C. Wang Z. Huang Z. Qin M. . (2023). Identification of colorectal cancer progression-associated intestinal microbiome and predictive signature construction. J. Transl. Med. 21, 373. doi: 10.1186/s12967-023-04119-1 37291572 PMC10249256

[B28] Liu S. Ma J. He F. (2024). A new SPQC biosensor for the detection of a new target of Escherichia/Shigella genera based on a novel method of synthesizing long-range DNA. Anal. Chem. 96, 9826–9833. doi: 10.1021/acs.analchem.4c00071 38829542

[B29] Maurelli A. T. Fernández R. E. Bloch C. A. Rode C. K. Fasano A. (1998). Black holes" and bacterial pathogenicity: a large genomic deletion that enhances the virulence of Shigella spp. and enteroinvasive Escherichia coli. Proc. Natl. Acad. Sci. U.S.A. 95, 3943–3948. doi: 10.1073/pnas.95.7.3943 9520472 PMC19942

[B19] Monge C. Waldrup B. Carranza F. G. Velazquez-Villarreal E. (2024). WNT and TGF-beta pathway alterations in early-onset colorectal cancer among Hispanic/Latino populations. Cancers (Basel) 16, 3903. doi: 10.3390/cancers16233903 39682092 PMC11639970

[B16] Nakayama M. Saito H. Murakami K. Oshima H. Oshima M. (2025). Missense mutant p53 transactivates Wnt/β-catenin signaling in neighboring p53-destabilized cells through the COX-2/PGE2 pathway. Cancer Res. Commun. 5, 13–23. doi: 10.1158/2767-9764.CRC-24-0471 39641656 PMC11695814

[B27] Older E. A. Zhang J. Ferris Z. E. Xue D. Zhong Z. Mitchell M. K. . (2023). Biosynthetic enzyme-guided disease correlation connects gut microbial metabolites sulfonolipids to inflammatory bowel disease involving TLR4 signaling. Nat Commun. 15 (1), 9371. doi: 10.1038/s41467-024-53670-y 39477928 PMC11525784

[B14] Phiri R. M. Ertuğrul M. B. Bozdoğan B. Hoşbul T. (2024). Evaluation of virulence genes in Proteus strains isolated from diabetic foot infections and urinary tract infections. J. Infect. Dev. Ctries 18, 1559–1565. doi: 10.3855/jidc.18928 39616486

[B1] Sadri S. Aghajani A. Soleimani H. Ghorbani Kalkhajeh S. Nazari H. Brouki Milan P. . (2024). Exploring the role of the TGF-β signaling pathway in colorectal precancerous polyps biochemical genetics. Biochem. Genet. 63, 1116–1148. doi: 10.1007/s10528-024-10988-y 39636332

[B10] Saraswat I. Goel A. (2024). Therapeutic modulation of the microbiome in oncology: current trends and future directions. Curr. Pharm. Bio/Technol. 26, 680–699. doi: 10.2174/0113892010353600241109132441 39543873

[B5] Sevcikova A. Martiniakova M. Omelka R. Stevurkova V. Ciernikova S. (2024). The link between the gut microbiome and bone metastasis. Int. J. Mol. Sci. 25, 12086. doi: 10.3390/ijms252212086 39596154 PMC11593804

[B4] Shah A. B. Baiseitova A. Zahoor M. Ahmad I. Ikram M. Bakhsh A. . (2024). Probiotic significance of Lactobacillus strains: a comprehensive review on health impacts, research gaps, and future prospects. Gut Microbes 16, 2431643. doi: 10.1080/19490976.2024.2431643 39582101 PMC11591481

[B11] Song Q. Gao Y. Liu K. Tang Y. Man Y. Wu H. (2024). Gut microbial and metabolomics profiles reveal the potential mechanism of fecal microbiota transplantation in modulating the progression of colitis-associated colorectal cancer in mice. J. Transl. Med. 22, 1028. doi: 10.1186/s12967-024-05786-4 39548468 PMC11566892

[B20] Su X. Wang X. Lai J. Mao S. Li H. (2024). Unraveling a novel hippo-associated immunological prognostic signature: the contribution of SERPINE1 in facilitating colorectal cancer progression via the notch signaling pathway. Genomics 116, 110794. doi: 10.1016/j.ygeno.2024.110794 38224823

[B34] Tran A. X. Trent M. S. Whitfield C. (2008). The LptA protein of Escherichia coli is a periplasmic lipid A-binding protein involved in the lipopolysaccharide export pathway. J. Biol. Chem. 283, 20342–20349. doi: 10.1074/jbc.M802503200 18480051 PMC2459282

[B31] Vacca M. Celano G. Calabrese F. M. Portincasa P. Gobbetti M. De Angelis M. (2020). The controversial role of human gut Lachnospiraceae. Microorganisms 8, 573. doi: 10.3390/microorganisms8040573 32326636 PMC7232163

[B26] Walker A. Pfitzner B. Harir M. Schaubeck M. Calasan J. Heinzmann S. S. . (2017). Sulfonolipids as novel metabolite markers of Alistipes and Odoribacter affected by high-fat diets. Sci. Rep. 7, 11047. doi: 10.1038/s41598-017-10369-z 28887494 PMC5591296

[B21] Wang P. Huang Q. Zhu Y. Chen L. Ye K. (2024). Fusobacterium nucleatum promotes microsatellite instability in colorectal carcinoma through up-regulation of miRNA-155-5p-targeted inhibition of MSH6 via the TLR4/NF-κB signaling pathway. Adv. Biol. (Weinh) 8, e2400293. doi: 10.1002/adbi.202400293 39334517

[B23] Wang Z. Dan W. Zhang N. Fang J. Yang Y. (2023). Colorectal cancer and gut microbiota studies in China. Gut Microbes 15, 2236364. doi: 10.1080/19490976.2023.2236364 37482657 PMC10364665

[B36] Xu X. Li J. Chua W. Z. Pages M. A. Shi J. Hermoso J. A. . (2023). Mechanistic insights into the regulation of cell wall hydrolysis by FtsEX and EnvC at the bacterial division site. Proc. Natl. Acad. Sci. U.S.A. 120, e2301897120. doi: 10.1073/pnas.2301897120 37186861 PMC10214136

[B22] Xu Y. Wu X. Li Y. Liu X. Fang L. Jiang Z. (2024). Probiotics and the role of dietary substrates in maintaining the gut health: use of live microbes and their products for anticancer effects against colorectal cancer. J. Microbiol. Biotechnol. 34, 1933–1946. doi: 10.4014/jmb.2403.03056 39210613 PMC11540615

[B37] Xu S. Zhang Y. Ding X. Yang Y. Gao J. Zou N. . (2025). Intestinal microbiota affects the progression of colorectal cancer by participating in the host intestinal arginine catabolism. Cell Rep. 44, 115370. doi: 10.1016/j.celrep.2025.115370 40022728

